# Pulmonary hemorrhage and associated risk factors among newborns admitted to a tertiary level neonatal unit in Botswana

**DOI:** 10.3389/fped.2023.1171223

**Published:** 2023-06-14

**Authors:** Alemayehu Mekonnen Gezmu, Endale Tefera, Kagiso Mochankana, Fizzah Imran, Dipesalema Joel, Irene Pelaelo, Britt Nakstad

**Affiliations:** ^1^ Department of Paediatrics and Adolescent Health, University of Botswana, Gaborone, Botswana; ^2^ Department of Paediatrics, Princess Marina Hospital Botswana MOH, Gaborone, Botswana; ^3^ Division of Paediatric and Adolescent Medicine, Inst Clinical Medicine, Faculty of Medicine, University of Oslo, Oslo, Norway

**Keywords:** pulmonary hemorrhage, premature newborns, risk factors, very low birth weight, extreme prematurity

## Abstract

**Introduction:**

Pulmonary hemorrhage (PH) is a life-threatening complication seen in very sick newborns with high morbidity and mortality. There is little data on the incidence, risk factors, and ultimate survival of newborns with pulmonary hemorrhage in sub-Saharan countries, where the healthcare provision and facility differ in many ways compared to high-income countries. Hence, this study aimed to determine the incidence, identify the risk factors, and describe the outcome of pulmonary hemorrhage in newborns in a low middle income country setting.

**Methods and materials:**

A cohort study with prospective data collection was conducted in a public, tertiary-level hospital in Botswana, the Princess Marina Hospital (PMH). All newborns admitted to the neonatal unit from 1 January 2020 to 31 December 2021 were included in the study. Data were collected using a checklist developed on the RedCap database (https//:ehealth.ub.ac.bw/redcap). The incidence rate of pulmonary hemorrhage was calculated as the number of newborns who had pulmonary hemorrhage per 1,000 newborns in the 2-year period. Group comparisons were made using *X*^2^ and Student’s *t*-tests. Multivariate logistic regression was used to identify risk factors independently associated with pulmonary hemorrhage.

**Result:**

There were 1,350 newborns enrolled during the study period, of which 729 were male newborns (54%). The mean (SD) birth weight was 2,154(±997.5) g, and the gestational age was 34.3 (±4.7) weeks. In addition, 80% of the newborns were delivered in the same facility. The incidence of pulmonary hemorrhage was 54/1,350 {4% [95% CI (3%–5.2%)]} among the newborns admitted to the unit. The mortality rate in those diagnosed with pulmonary hemorrhage was 29/54 (53.7%). Multivariate logistic regression identified birth weight, anemia, sepsis, shock, disseminated intravascular coagulopathy (DIC), apnea of prematurity, neonatal encephalopathy, intraventricular hemorrhage, mechanical ventilation, and blood transfusion as risk factors independently associated with pulmonary hemorrhage.

**Conclusion:**

This cohort study identified a high incidence and mortality rate of pulmonary hemorrhage in newborns in PMH. Multiple risk factors, such as low birth weight, anemia, blood transfusion, apnea of prematurity, neonatal encephalopathy, intraventricular hemorrhage, sepsis, shock, DIC, and mechanical ventilation, were identified as independently associated risk factors for PH.

## Introduction

Pulmonary hemorrhage (PH) is diagnosed when a discharge of large amounts of bloody fluid from the endotracheal tube or respiratory tract is identified in very sick newborns who have shown acute clinical deterioration (1, 2). PH is one of the most acute and feared causes of mortality in newborns. The mortality from PH ranges from 50% to 68%, and the PH incidence rate is reported to be 1–2 per 1,000 live births occurring most commonly within the first few days of life (2, 3). PH results in an acute deterioration reflected by respiratory compromise, hemodynamic instability, and the need for ventilatory support (4).

Numerous studies have identified multiple risk factors for PH. A higher incidence of PH was observed in premature newborns with extreme prematurity and very low birth weight, as compared to newborns with higher birth weights and gestational ages (GA) (1). The literature identifies risk factors for PH as intrauterine growth restriction, respiratory distress syndrome (RDS), lung hypoplasia, sepsis, coagulopathy, thrombocytopenia, intraventricular hemorrhage (IVH), patent ductus arteriosus (PDA), ventilator treatment, and surfactant treatment (3, 5, 6).

Understanding the risk factors and identifying high-risk patients is critical for devising effective preventive and treatment measures as this would help to mitigate the high mortality and morbidity associated with PH in newborns. In a systematic review, ventilatory support, epinephrine administration, management of coagulopathy, and administration of tolazoline were all found to be effective primary treatments for PH (2).

This study aimed to determine the incidence of PH and identify risk factors associated with pulmonary hemorrhage in newborns admitted at the largest tertiary-level, public hospital in Botswana through a 2-year period.

## Methods and materials

This cohort study with prospective data collection was conducted in the Princess Marina Hospital (PMH) in Gaborone, Botswana, where predominantly those with low middle income and socioeconomic status are admitted, whereas those with higher socioeconomic status or health insurance, in our experience, deliver in private hospitals. The unit has 38 beds including six neonatal intensive care beds and admits approximately 100 newborns in a month. Most of the admitted newborns are born in the facility, some are referred from local clinics and primary- or secondary-level hospitals around the capital city and southern part of Botswana. The unit provides basic and advanced clinical care for newborns with all types of medical conditions, except for advanced surgery and extracorporeal membrane oxygenation (ECMO). Due to high mortality rates, especially among the smallest, the unit provides intensive care for babies with birth weight >900 g or GA >28 weeks. All newborns admitted to the neonatal unit were enrolled if the mother gave spoken and written consent to allow the collection of clinical data. The recruitment period was from 1 January 2020 to 31 December 2021. Data were collected using a checklist developed on the RedCap database (https//:ehealth.ub.ac.bw/redcap). The data were collected by a pediatrics registrar, consultant pediatrician, and neonatologist on a daily basis for 10 days and then weekly for each admitted newborn until the occurrence of end points: discharge, death, or transfer out from the unit.

### Statistical analysis

The sample size was calculated based on one sample population proportion taking the incidence of pulmonary hemorrhage at 10% from studies done in South Africa (7). Assuming that 10% of the neonates admitted in the unit have pulmonary hemorrhage, and a finite population size of 1,400 neonates admitted in the unit per year, with a 95% level of confidence and with absolute precision of 10%, the study requires a sample size of 997. After looking into the number of newborns admitted to our study, we decided to include all 1,350 study participants in the analysis.

The collected data were exported to SPSS version 27 for Mac (IBM, Chicago, United States) for analysis. Descriptive statistics were presented as frequency and percentage for categorical variables and mean and standard deviation (SD) for continuous variables. The incidence rate was calculated as a proportion with absolute precision 95% confidence interval. Group comparisons were made using *X*^2^ and Student’s *t*-tests for categorical and continuous variables, respectively. A multivariate logistic regression model was used to identify risk factors independently associated with pulmonary hemorrhage. Statistical significance was set at a *P*-value of <0.05.

### Operational definitions

The following operational definitions were used.

*Pulmonary hemorrhage in the newborn* was defined when a discharge of large amounts of bloody fluid from the endotracheal tube or respiratory tract occurred in very sick newborns who showed acute clinical deterioration like blood aspirated from the trachea concurrent with respiratory decompensation that necessitated intubation or escalated support. When traces of blood appeared at suctioning secretions from the respiratory tract, this was not defined as PH.

*Anemia* was observed prior to PH in this study and according to the definitions in the unit’s protocol. Early (first 1–2 days) anemia was Hb (venous or arterial) < 13.5 g/dl. Later anemia, also before PH happened, depended on the day of life (DOL) and gestational age.

*Transfusion of red blood cells* was performed before PH occurred when Hb level is <11–12 g/dl and the need for respiratory support, including the need for supplemental oxygen.

*Apnea* was defined as the absence of breathing/cessation of respiratory airflow in a neonate for a period of >15–20 s often associated with bradycardia and/or desaturation.

*Presumed sepsis* was defined as the presence of non-specific clinical signs and symptoms consistent with sepsis and therefore starting treatment with antibiotics. Symptoms and signs were any five of the following: difficulty breathing, apnea, increased mean respiratory rate of more than two standard deviations (SDs) above normal for age or the need for mechanical ventilation for an acute process (not related to underlying neuromuscular disease or the need for general anesthesia), temperature lability (core temperature >38.5° or <36°C), low blood pressure and poor perfusion, abdominal distention, hyperglycemia, bloody stools, or convulsions. Abnormal whole blood cell counts and immature neutrophils were part of the evaluation for presumed sepsis.

*Proven sepsis* was defined when presumed sepsis and if the blood culture was positive.

*Septic shock* was defined if the condition required vasopressor treatment if presumed or proven sepsis.

*Disseminated intravascular coagulation (DIC)* was suspected and defined if simultaneous bleeding and clotting occurred during blood sampling.

*PDA* was confirmed by echocardiographic evaluation of each newborn when clinically suspected of having PDA, performed by a pediatric cardiologist.

*Persistent pulmonary hypertension of the newborn (PPHN)* in our setting is diagnosed by echocardiography. In PPHN, echocardiography demonstrates normal structural cardiac anatomy with evidence of pulmonary hypertension [i.e., elevated right ventricle pressure (RVp)]. RVp can be estimated based on Doppler measurement of the velocity of the tricuspid regurgitation (TR) jet, if present. If there is no TR, RVp can be assessed qualitatively (e.g., flattened or displaced ventricular septum).

*Transient tachypnea* is a mild, self-limited respiratory problem in neonates that begins after birth and lasts about 3 days, also named “wet lungs” or type II respiratory distress syndrome, often without carbon dioxide retention.

## Results

There were 1,350 newborns enrolled during the study period, of which 729 (54%) were male newborns. The mean (SD) birth weight was 2,154 (±997.5) g, and the gestational age was 34.3 (±4.7) weeks. In addition, 80% of the newborns were delivered in PMH. Other deliveries were home deliveries or born at clinics outside PMH (Table 1).

**Table 1:** Demographic and baseline characteristics of newborns admitted to the neonatal care unit.

Variables	*n* (%)
Sex	
Male	729 (54.0)
Female	621 (46.0)
Gestational age (weeks)	
<32	412 (30.5)
≥32	938 (69.5)
Mean (SD)	34.3 (4.7)
Birth weight (grams)	
<1,500 (VLBW)	443 (32.8)
<1,000 g (ELBW)	122 (9.0)
≥1,500	907 (67.2)
Mean (SD)	2,154.3 (997.5)
Location of birth	
Inborn	1,081 (80.2)
Home delivery	30 (2.2)
Other health facilities	237 (17.6)
Admission diagnosis	
RDS	570 (42)
Congenital pneumonia	103 (7.6)
BPD	155 (11.5)
TTN	144 (10.7)
MAS	72 (5.3)
PPHN	34 (2.5)
Anemia	91 (6.7)
Laboratory-confirmed bloodstream infection (sepsis)	101 (7.5)
Clinically suspected sepsis	886 (65.6)
Shock	46 (3.4)
DIC	10 (0.7)
Pulmonary hemorrhage	54 (4.0)
Neonatal jaundice	469 (34.7)
Neonatal encephalopathy	87 (6.4)
HIE	99 (7.4)
IVH	58 (4.1)
Patent ductus arteriosus	52 (3.9)
Apgar score <5 at 5 min	
Yes	217 (16.1)
No	1,117 (82.7)

VBLW, very low birth weight; ELBW, extremely low birth weight infants; RDS, respiratory distress syndrome; BPD, bronchopulmonary dysplasia; TTN, transient tachypnea of the newborn; MAS, meconium aspiration syndrome; PPHN, persistent pulmonary hypertension of the newborn; DIC, disseminated intravascular coagulopathy; HIE, hypoxic ischemic encephalopathy; IVH, intraventricular hemorrhage.

Table 1 also depicts clinically suspected sepsis [886 (65.6%)], RDS [570 (42%)], and neonatal jaundice [469 (34.7%)] that were the most common clinical diagnoses observed in the admitted newborns.

The incidence of pulmonary hemorrhage among the newborns admitted to our unit was 54/1,350 {4% [95% CI (3–5.2%)]}. The incidence of PH in very low birth weight (VLBW) newborns was 2.96%. The mortality rate among all newborns diagnosed with pulmonary hemorrhage was 29/54 (53.7%). Variables, such as gestational age, birth weight, RDS, bronchopulmonary dysplasia (BPD), sepsis, Apgar score at 5 min <5, and mechanical ventilation, were included in univariate analysis. On univariate analysis, all entered variables were statistically significant at *P*-value <0.05 (Table 2). Variables with *P*-value <0.1 were included for multivariate logistic regression analysis. Of these variables, birth weight [aOR (95% CI) 0.85 (0.77–0.94), *P* = 0.002], anemia [aOR (95% CI) 3.35 (1.4–7.66) *P* = 0.004], sepsis [aOR (95% CI) 2.62 (1.10–6.24), *P* = 0.029], and shock [aOR (95% CI) 5.78 (1.82–18.38), *P* = 0.003] were independently associated with risk of pulmonary hemorrhage (Table 2).

**Table 2:** Factors associated with pulmonary hemorrhage in newborns admitted to the neonatal care unit.

Variables	Pulmonary hemorrhage, *n* (%)	No pulmonary hemorrhage, *n* (%)	Unadjusted OR (95% CI)	*P*-value	Adjusted OR (95% CI)	*P*-value
Sex						
Male	27 (3.7)	702 (96.3)	1.18 (0.69–2.04)	0.547		
Female	27 (4.3)	594 (95.7)				
Gestational age (weeks)						
Mean (SD)	30.54 (3.88)	34.42 (4.6)	0.83 (0.78–0.88)	<0.001	1 (0.99–1.00)	0.505
Birth weight (g)						
Mean (SD)	1,422.4 (756.6)	2,184.8 (994.8)	1 (0.98–0.99)	<0.001	0.85 (0.77–0.94)	**0** **.** **002**
Clinical diagnosis						
Respiratory distress syndrome	43 (7.5)	527 (92.5)	5.7 (2.92–11.26)	<0.001	1.16 (0.39–3.44)	0.785
Bronchopulmonary dysplasia	12 (7.7)	143 (92.3)	2.30 (1.19–4.48)	0.012	1.62 (0.60–4.37)	0.340
Transient tachypnea of the newborn	1 (0.7)	143 (99.3)	0.15 (0.02–1.11)	0.032	0.66 (0.07–6.34)	0.720
PPHN	4 (11.8)	30 (88.2)	3.38 (1.15–9.95)	0.044	1.84 (0.26–13.22)	0.543
Anemia	20 (22.0)	71 (78.0)	10.15 (5.56–18.53)	<0.001	3.35 (1.4–7.66)	**0**.**004**
Sepsis	11 (10.9)	90 (89.1)	3.43 (1.71–6.88)	0.001	2.62 (1.10–6.24)	**0**.**029**
Clinically suspected sepsis	45 (5.1)	841 (94.9)	2.71 (1.31–5.58)	0.005	2.33 (0.73–7.46)	0.153
Shock (septic)	13 (28.3)	33 (71.7)	12.14 (5.95–24.76)	<0.001	5.78 (1.82–18.38)	**0**.**003**
DIC	5 (50.0)	5 (50.0)	26.35 (7.38–94.00)	<0.001	78.15 (5.90–1,035.01)	**0**.**001**
Neonatal jaundice	30 (6.4)	439 (93.6)	2.44 (1.41–4.23)	0.001	1.25 (0.58–5.78)	0.353
Patent ductus arteriosus	7 (13.5)	45 (86.5)	4.14 (1.77–9.67)	0.004	1.47 (0.65–3.30)	0.149
Apnea of prematurity	20 (16.5)	101 (83.5)	6.96 (3.86–12.54)	<0.001	2.30 (0.74–7.14)	**0**.**033**
Neonatal encephalopathy	8 (9.2)	79 (90.8)	2.68 (1.22–5.87)	0.019	2.50 (1.08–5.79)	**0**.**001**
IVH grade 2	4 (36.4)	7 (63.6)	14.73 (4.18–51.96)	<0.001	7.67 (2.26–25.98)	**0**.**050**
IVH grade 3	4 (20.0)	16 (80.0)	6.40 (2.06–19.84)	0.007	4.86 (0.99–23.94)	0.775
Apgar score <5 at 5 min	17 (7.8)	202 (92.2)	0.40 (0.22–0.73)	0.002	1.05 (0.40–2.70)	0.928
Mode of ventilation						
Spontaneous	17 (1.7)	955 (98.3)				**0**.**005**
Mechanical ventilation	29 (14.2)	175 (85.5)	2.54 (1.83–5.53)	<0.001	3.33 (1.43–7.79)	
CPAP	8 (7.3)	102 (92.7)				
Bradycardia needing mask/bag ventilation						
Yes	12 (14.8)	69 (85.2)	4.75 (2.39–9.42)	<0.001	1.47 (0.42–5.07)	0.547
Blood product transfusion						
Transfused	6 (28.6)	15 (71.4)	9.16 (3.37–24.92)	<0.001	4.21 (1.15–15.43)	**0**.**030**

SD, standard deviation; CPAP, continuous positive airway pressure; DIC, disseminated intravascular coagulation; IVH, intraventricular hemorrhage; PPHN, persistent pulmonary hypertension of the newborn.

The bold numbers indicate statistical difference.

## Discussion

This study found a 4% high incidence rate of PH in admitted newborns at the largest tertiary hospital in Botswana and about 54% mortality rate in those diagnosed with PH. This incidence and mortality rate was similar to other studies (1, 2, 8). However, there is substantial variation in reported PH incidence and associated mortality rates between high-income countries (HICs) and low middle income countries (LMICs). In a systematic review from HIC in 2012, the incidence of PH was 1–12 per 1,000 live births (9), whereas a study from China reported PH in premature newborns of BW <1,500 g at 6.1% and BW <1,000 g at 22.9%, respectively (1). Also from China, a higher neonatal mortality rate was reported in those with PH and a birth weight less than 1,500 g, 82.1% (96/117), and 67.7% (86/127) in those with a birth weight above 1,500 g (10). In a large retrospective cohort study from multiple neonatal intensive care units in the USA, mortality rates among patients with PH were 40.6% and 54.0% at 7 and 30 days of age, respectively (11).

PH can start gradually and continue for a long time, or it can be a sudden life-threatening respiratory complication associated with several risk factors (12). We identified multiple risk factors to be independently associated with pulmonary hemorrhage. In our cohort low birth weight, anemia, blood transfusion, apnea of prematurity, neonatal encephalopathy, intraventricular hemorrhage, sepsis, shock, DIC, and mechanical ventilation were all independent risk factors for PH. According to the literature (13), bleeding into the lungs occurs mainly in premature newborns with severe lung disease. Risk factors have been proposed to be respiratory problems (14) and hemodynamically significantly increased blood flow (persistent ductus arteriosus, PDA) in the blood vessels in the lungs (15), like when sepsis impacts on hemodynamics and reopens or enlarges the patent ductus arteriosus. In newborns, infection (8), in accordance with our findings of sepsis and shock being highly associated with PH, as well as the association of PH with apnea of prematurity (16), is in line with the results of our study. Our preterm babies that developed PH were critically ill with clinical conditions like sepsis or neonatal encephalopathy. A retrospective study that reported on perinatal risk factors from China identified a rate of PH in extremely low birth weight infants (ELBW, <1,000 g) to be 18.8%. These preterm babies that experienced PH were critically ill with clinical conditions like sepsis or neonatal encephalopathy. It has been shown that small for gestational age, early onset neonatal sepsis, low birth weight, lower Apgar scores at 1 and 5 min, severe RDS, and surfactant replacement were all risk factors for PH (12). Another study from a neonatal intensive care unit in Brazil reported prior use of blood components to be an independent risk factor for PH (17). Transfusion-related PH and acute illness may be more common than previously thought (18, 19). The evidence from preclinical studies showed that blood products can directly modulate transfusion-related immunomodulation, a mechanism linking transfusion exposure with neonatal morbidities (20). Additionally, the use of blood products prior to the PH episode may cause a sudden increase in blood volume, leading to stress injury of the capillary wall, with the passage of fluid and plasma proteins, which can also lead to left ventricular failure, contributing to an increase in pulmonary capillary blood pressure (17). Preterm newborns represent one of the most frequently transfused patients (21). In accordance with our findings, a large retrospective study in preterm neonates found that those who received any RBC transfusion had a 50% increase in neonatal mortality (20).

In our cohort of 1,350 newborns, we found that recurrent apnea was highly correlated to PH. Apnea is a common condition in premature infants due to the immaturity of respiratory control mechanisms. Recurrent apnea is associated with respiratory failure, pulmonary hemorrhage, abnormal heart and lung function, intracranial hemorrhage, abnormal nervous system development, and even sudden death (22). Studies in infants indicated that negative pressure pulmonary edema because of upper airway obstruction led to pulmonary hemorrhage thought to be a stress failure caused by high negative intrathoracic pressure and mechanical disruption of the alveolar-capillary membrane leading to pulmonary bleeding (23). There could be a similar mechanism to apnea causing PH due to laryngospasm or high negative pleural pressure due to inspiratory effort during upper airway obstruction (24, 25).

In newborns, pulmonary hemorrhage is often a manifestation of pulmonary edema (26), and PDA (5) can range in severity from blood-tinged secretions in the endotracheal tube to life-threatening blood loss with hypovolemic shock. The cause of PH is thought to be due to the rapid lowering of intrapulmonary pressure, which facilitates left-to-right shunting across a PDA and an increase in pulmonary blood flow (27). In our study, we identified PDA as a risk factor for PH with statistical significance on univariate analysis, but on multivariate analysis, we could not show statistical significance.

This study has several limitations. First, this study only included study participants from a single neonatal center. Second, important variables such as the use of surfactant were not captured to give light to the impact of surfactant treatment on the incidence and outcome of PH. Finally, because of the policy of the unit, only ELBW infants of 900–1,000 g were provided intensive care and respiratory support whereas smaller ELBWs (<900 g) were provided nasal oxygen treatment, enteral and parenteral nutrition, and antibiotic treatment. We could not analyze and discuss the outcome of those ELBWs (BW < 900 g) and extremely premature babies based on the treatment provided.

## Conclusion

This cohort study identified a high incidence and mortality rate in neonates with pulmonary hemorrhage. Multiple risk factors, such as low birth weight, anemia, blood transfusion, apnea of prematurity, neonatal encephalopathy, intraventricular hemorrhage, sepsis, shock, DIC, and mechanical ventilation, were identified as independently associated risk factors for PH.

## Data Availability

The raw data supporting the conclusions of this article will be made available by the authors, without undue reservation.

## References

[1] Li J Xia H Ye L Li X Zhang Z. Exploring prediction model and survival strategies for pulmonary hemorrhage in premature infants: a single-center, retrospective study. Transl Pediatr. (2021) 10(5):1324. 10.21037/tp-21-64 34189090 PMC8193000

[2] Barnes ME Feeney E Duncan A Jassim S MacNamara H O’Hara J Pulmonary haemorrhage in neonates: systematic review of management. Acta Paediatr. (2022) 111(2):236–44. 10.1111/apa.16127 34582587

[3] Usemann J Garten L Bührer C Dame C Cremer M. Fresh frozen plasma transfusion–a risk factor for pulmonary hemorrhage in extremely low birth weight infants? J Perinat Med. (2017) 45(5):627–33. 10.1515/jpm-2016-0309 28195553

[4] Bozdağ Ş Dilli D Gökmen T Dilmen U. Comparison of two natural surfactants for pulmonary hemorrhage in very low-birth-weight infants: a randomized controlled trial. Am J Perinatol. (2015) 32(03):211–8. 10.1055/s-0034-1389090 25241106

[5] Kluckow M Evans N. Ductal shunting, high pulmonary blood flow, and pulmonary hemorrhage. J Pediatr. (2000) 137(1):68–72. 10.1067/mpd.2000.106569 10891824

[6] Yen T-A Wang C-C Hsieh W-S Chou H-C Chen C-Y Tsao P-N. Short-term outcome of pulmonary hemorrhage in very-low-birth-weight preterm infants. Pediatr Neonatol. (2013) 54(5):330–4. 10.1016/j.pedneo.2013.04.005 23711674

[7] Sankar MJ Gupta N Jain K Agarwal R Paul VK. Efficacy and safety of surfactant replacement therapy for preterm neonates with respiratory distress syndrome in low- and middle-income countries: a systematic review. J Perinatol. (2016) 36(1):S36–48. 10.1038/jp.2016.31 27109091 PMC4848743

[8] Lin T-W Su B-H Lin H-C Hu P-S Peng C-T Tsai C-H Risk factors of pulmonary hemorrhage in very-low-birth-weight infants: a two-year retrospective study. Acta Paediatr Taiwanica Taiwan er ke yi xue hui za zhi. (2000) 41(5):255–8. 11100523

[9] Zahr RA Ashfaq A Marron-Corwin M. Neonatal pulmonary hemorrhage. Neoreviews. (2012) 13(5):e302–6. 10.1542/neo.13-5-e302

[10] Li L Yu J Wang J Zhang X Shen H Yuan X A prediction score model for risk factors of mortality in neonate with pulmonary hemorrhage: the experience of single neonatal intensive care unit in southwest China. Pediatr Pulmonol. (2008) 43(10):997–1003. 10.1002/ppul.20897 18785623

[11] Ahmad KA Bennett MM Ahmad SF Clark RH Tolia VN. Morbidity and mortality with early pulmonary haemorrhage in preterm neonates. Arch Dis Childhood Fetal Neonatal Ed. (2019) 104(1):F63–8. 10.1136/archdischild-2017-314172 29374627

[12] Wang T-T Zhou M Hu X-F Liu J-Q. Perinatal risk factors for pulmonary hemorrhage in extremely low-birth-weight infants. World J Pediatr. (2020) 16(3):299–304. 10.1007/s12519-019-00322-7 31686366 PMC7312118

[13] Tomaszewska M Stork E Minich NM Friedman H Berlin S Hack M. Pulmonary hemorrhage: clinical course and outcomes among very low-birth-weight infants. Arch Pediatr Adolesc Med. (1999) 153(7):715–21. 10.1001/archpedi.153.7.715 10401804

[14] Wang L Zhao L Xu J Yu Y Li Z Zhang F Association between pulmonary hemorrhage and CPAP failure in very preterm infants. Front Pediatr. (2022) 10:938431. 10.3389/fped.2022.938431 PMC9500376 36160772

[15] Kappico JM Cayabyab R Ebrahimi M Uzunyan MY Barton L Siassi B Pulmonary hemorrhage in extremely low birth weight infants: significance of the size of left to right shunting through a valve incompetent patent foramen ovale. J Perinatol. (2022) 42(9):1233–7. 10.1038/s41372-022-01464-9 35851183

[16] Ayebare E Hanson C Nankunda J Hjelmstedt A Nantanda R Jonas W Factors associated with birth asphyxia among term singleton births at two referral hospitals in northern Uganda: a cross sectional study. BMC Pregnancy Childbirth. (2022) 22(1):1–12. 10.1186/s12884-022-05095-y 36224532 PMC9559004

[17] Ferreira CH Carmona F Martinez FE. Prevalence, risk factors and outcomes associated with pulmonary hemorrhage in newborns. J Pediatr (Rio J). (2014) 90:316–22. 10.1016/j.jped.2013.12.008 24606947

[18] Sanchez R Toy P. Transfusion related acute lung injury: a pediatric perspective. Pediatr Blood Cancer. (2005) 45(3):248–55. 10.1002/pbc.20395 15852432

[19] Valentine SL Cholette JM Goobie SM. Transfusion strategies for hemostatic blood products in critically ill children: a narrative review and update on expert consensus guidelines. Anesth Analg. (2022) 135(3):545–57. 10.1213/ANE.0000000000006149 35977364

[20] dos Santos AMN Guinsburg R de Almeida MFB Procianoy RS Leone CR Marba STM Red blood cell transfusions are independently associated with intra-hospital mortality in very low birth weight preterm infants. J Pediatr. (2011) 159(3):371–6. 10.1016/j.jpeds.2011.02.040 21489555

[21] Strauss RG. Red blood cell transfusion practices in the neonate. Clin Perinatol. (1995) 22(3):641–55. 10.1016/S0095-5108(18)30273-2 8521686

[22] Chen J Jin L Chen X. Efficacy and safety of different maintenance doses of caffeine citrate for treatment of apnea in premature infants: a systematic review and meta-analysis. Biomed Res Int. (2018) 2018:9061234. 10.1155/2018/9061234 30671477 PMC6323495

[23] Schwartz DR Maroo A Malhotra A Kesselman H. Negative pressure pulmonary hemorrhage. Chest. (1999) 115(4):1194–7. 10.1378/chest.115.4.1194 10208229

[24] Dolinski SY MacGregor DA Scuderi PE. Pulmonary hemorrhage associated with negative-pressure pulmonary edema. J Am Soc Anesthesiol. (2000) 93(3):888–90. 10.1097/00000542-200009000-00042 10969326

[25] Bersten AD Patel AR. Pulmonary haemorrhage associated with negative-pressure pulmonary oedema: a case report. Crit Care Resusc. (2006) 8(2):115–6. 10.3316/informit.517611976385693 16749876

[26] Cole VA Normand ICS Reynolds EOR Rivers RPA. Pathogenesis of hemorrhagic pulmonary edema and massive pulmonary hemorrhage in the newborn. Pediatrics. (1973) 51(2):175–87. 10.1542/peds.51.2.175 4695850

[27] Aziz A Ohlsson A. Surfactant for pulmonary haemorrhage in neonates. Cochrane Database Syst Rev. (2020) 2(2):CD005254. 10.1002/14651858.CD005254.pub4 PMC6996938 32012227

